# Sleep Difficulties and Cognition for 10 Years in a National Sample of U.S. Older Adults

**DOI:** 10.1093/geroni/igaa025

**Published:** 2020-06-29

**Authors:** Rebecca Robbins, Amanda Sonnega, Robert W Turner, Girardin Jean-Louis, Mark Butler, Ricardo S Osorio, Kenneth M Langa

**Affiliations:** 1 Division of Sleep and Circadian Disorders, Brigham & Women’s Hospital, Boston, Massachusetts; 2 Harvard Medical School, Boston, Massachusetts; 3 Survey Research Center Institute for Social Research, The University of Michigan, Ann Arbor; 4 Department of Clinical Research and Leadership, The George Washington University School of Medicine and Health Sciences, District of Columbia; 5 Center for Healthful Behavior Change, Department of Population Health, NYU Langone Health; 6 Department of Psychiatry, NYU Langone Health; 7 Center for Brain Health, NYU Langone Health; 8 Department of Internal Medicine, Institute for Social Research, Institute for Healthcare Policy and Innovation, Veterans Affairs Ann Arbor Healthcare System, University of Michigan

**Keywords:** Cognitive function, Gerontology, Healthy aging, Sleep, Translational medicine

## Abstract

**Background and Objectives:**

Sleep difficulties are common among older adults and are associated with cognitive decline. We used data from a large, nationally representative longitudinal survey of adults aged older than 50 in the United States to examine the relationship between specific sleep difficulties and cognitive function over time.

**Research Design and Methods:**

Longitudinal data from the 2004–2014 waves of the Health and Retirement Study were used in the current study. We examined sleep difficulties and cognitive function within participants and across time (*n* = 16 201). Sleep difficulty measures included difficulty initiating sleep, nocturnal awakenings, early morning awakenings, and waking up feeling rested from rarely/never (1) to most nights (3). The modified Telephone Interview for Cognitive Status was used to measure cognitive function. Generalized linear mixed models were used with time-varying covariates to examine the relationship between sleep difficulties and cognitive function over time.

**Results:**

In covariate-adjusted models, compared to “never” reporting sleep difficulty, difficulty initiating sleep “most nights” was associated with worse cognitive function over time (Year 2014: *b* = −0.40, 95% CI: −0.63 to −0.16, *p* < .01) as was difficulty waking up too early “most nights” (Year 2014: *b* = −0.31, 95% CI: −0.56 to −0.07, *p* < .05). In covariate-adjusted analyses, compared to “never” reporting waking up feeling rested, cognitive function was higher among those who reported waking up feeling rested “some nights” (Year 2010: *b* = 0.21, 95% CI: 0.02 to 0.40, *p* < .05).

**Discussion and Implications:**

Our findings highlight an association between early morning awakenings and worse cognitive function, but also an association between waking up feeling rested and better cognitive function over time.

**Translational Significance:** Sleep difficulties are common among older adults yet reduce the quality of life and also contribute to the development of and potentially accelerate cognitive decline. This study examines specific sleep difficulties (e.g., difficulty falling asleep) and their unique relationship to cognition over time among older adults in the United States. The primary aim of this work is to illuminate the specific sleep difficulties that are most concerning from the standpoint of cognitive impairment so as to inform the design of future tailored sleep improvement programs for older adults.

Sleep difficulties (e.g., difficulty initiating sleep and early morning awakenings) are more common among older adults (65 years or older) than any other age group in the United States (1). Particularly concerning is that sleep difficulties are associated with adverse outcomes. A review of 85 studies assessing sleep difficulty among older adults showed that sleep difficulties were detrimental to physical health and psychosocial well-being (2). Furthermore, a growing body of evidence suggests a connection between sleep difficulties and cognitive impairment and the development and progression of Alzheimer’s disease (AD). With an aging population, it is critical to identify factors, such as sleep difficulty, that contribute to the initiation and worsening of AD and related dementias (3).

Research has examined sleep and cognition among older adults and employed a range of sleep parameters in doing so. In research conducted among a nationally representative sample of men using both objective and self-reported measures, researchers found a proxy for sleep difficulty (i.e., self-reported poor sleep quality) and objectively recorded nighttime awakenings were associated with cognitive decline in the follow-up period, which was between 3 and 5 years (4). Another nationally representative study using both objective and self-reported measures did not find any significant associations between self-reported sleep difficulties and cognition during the 5 years, but researchers did find objective recordings of sleep fragmentation and waking after sleep onset were associated with cognitive decline (5). Results from cross-sectional analyses showed a significant association between insomnia (i.e., chronic sleep difficulty) and reduced performance in learning and temporal order judgment among older adults (6). Similarly, another study found a measure similar to sleep difficulty (fragmented sleep) was a significant predictor of cognitive impairment at follow-up (which averaged 3 years) (7). Finally, a meta-analysis of cross-sectional and prospective studies, broadly defined and inclusive of components such as sleep quality, insomnia, and sleep apnea, found sleep difficulty was associated with cognitive impairment among older adults (8).

The literature to date is limited in several ways. One clear critique of the literature is that the conceptualization of sleep difficulty has ranged widely from one study to another. Indeed, as noted above, research has employed a range of sleep difficulty measures, ranging from sleep apnea to daytime sleepiness. Furthermore, existing literature often develops summary scores for sleep difficulty as opposed to measures of specific sleep difficulties (i.e., difficulty initiating sleep, nocturnal awakenings, and early morning awakenings) (8,9). Also, much of the evidence is based upon either cross-sectional designs or prospective observational study designs with short follow-up periods. Moreover, to date, no longitudinal studies have examined associations of sleep difficulty (i.e., difficulty initiating sleep, nocturnal awakenings, early morning awakenings, or waking not feeling rested) with cognitive function using national data.

The present study addressed these gaps in the literature by examining specific symptoms of sleep difficulty across time and their association to cognitive function using 10 years of longitudinal, national data from the Health and Retirement Study (HRS). We hypothesized that each sleep difficulty (i.e., difficulty initiating sleep, nocturnal awakenings, early morning awakenings, or not waking up feeling rested) will be associated with worse cognitive function over time in covariate-adjusted analyses.

## Method

We analyzed data from the HRS, an ongoing nationally representative longitudinal survey of adults aged older than 50 in the United States. The study began in 1992, with a core interview administered every 2 years. In 1998, HRS instituted a steady-state design, enrolling a new birth cohort every 6 years. The Health and Retirement Study uses a multistage area probability sampling design that includes geographic stratification, clustering, and oversampling of African American, Hispanic, and minority households (10). During each biennial wave of data collection, approximately 20,000 participants are surveyed. The HRS also uses proxy respondents for those individuals who are unwilling or unable to complete an interview, which has been shown to reduce attrition bias in longitudinal studies (11). Additional information regarding the study design and content is available elsewhere (12). For most variables, we used a clean and ready-to-use version of the data (13). All respondents provided consent, and the study protocol was approved by the University of Michigan Institutional Review Board.

### Analytic Sample

We began with 18,327 respondents who participated in the 2004 wave of the HRS. While the sleep difficulty variables were available beginning in the prior wave in 2002, we chose 2004 as our baseline in order to maximize our sample for longitudinal analysis because 2004 was the enrollment year for the Early Baby Boomer cohort (individuals born between the years 1948 and 1953). We selected 17 592 respondents who demonstrated no significant signs of cognitive impairment at baseline, as evidenced by a score of 7 or higher on the validated Telephone Interview for Cognitive Status (TICS-m) during the baseline year for this analysis (2004) representing 96.0% of respondents. We then selected 16,494 respondents (90.0% of the population) who were aged older than 50 years. We excluded those respondents who were represented by a proxy (*n* = 293). The final analytic sample was 16,201 respondents (88.4% of the population). The Health and Retirement Study collected information on sleep difficulties biennially until 2006, then in alternate waves thereafter. A new birth cohort was enrolled in 2010, but we excluded these new participants from our sample to maintain a consistent follow-up interval for all respondents. Thus, our analytic sample included all respondents surveyed in 2004 and at follow-up (Years 2006, 2010, and 2014).

### Measures

#### Sleep difficulty

Sleep difficulty was measured with four items. Participants were asked to report the frequency of the following: (i) difficulty initiating sleep, (ii) difficulty maintaining sleep (nocturnal awakenings), (iii) early morning awakenings, and (iv) waking up feeling rested. Response options included 1 (most nights), 2 (sometimes), and 3 (rarely/never). We reverse-scored the items so that higher scores indicated more sleep difficulty (1 = rarely/never, 2 = sometimes, and 3 = most nights) with the exception of feeling rested, for which higher values indicated more feelings of being rested. Finally, we created a new variable to indicate those who experienced concurrent sleep difficulties. Specifically, we identified those individuals who responded either “most nights” to two or more difficulties in a single wave (i.e., difficulty initiating sleep, maintaining sleep, or early morning awakenings) or “rarely/never” to the question regarding waking up and feeling refreshed.

#### Cognitive function

Cognitive function was measured using the TICS-m, which included immediate and delayed recall items, serial 7s subtraction, and counting backward. Immediate list recall included presenting 10 English nouns audibly to participants at a 2 seconds/word rate (14). Participants were then asked to recall the words in any order they could remember orally, and the examiner recorded their responses. Serial 7s included a serial subtraction by 7s from 100 for five trials. This task taps numerical ability and working memory or the ability to hold and transform information simultaneously. The modified Telephone Interview for Cognitive Status has been used to screen for dementia and mild cognitive impairment. The total cognitive score was measured continuously using the composite measure, which ranged from 0 to 27. As a reference, a score ranging from 0 to 6 is consistent with dementia, 7–11 is consistent with cognitive impairment without dementia, and 12–27 is considered normal (14,15).

#### Covariates

We controlled for a number of covariates that are known to be associated with cognitive function (13) and sleep difficulties (16). Several covariates were taken from the respondent’s baseline interview including age in years; gender coded as 1 = female, 0 = male; race coded as 1 = white, 0 = nonwhite; ethnicity coded as 1 = Hispanic, 0 = non-Hispanic; marital status coded as 1 = married or living with a partner, 0 = unmarried (never married, widowed, and divorced); education coded as years of schooling (0–17 years or more); and total self-reported financial assets coded by quartile (1 = <$55,000, 2 = $55,000 to $182,999, 3 = $183,000 to $464,999, and 4 = $465,000 or higher). For all other covariates, we included wave-specific measures. Chronic medical conditions were a count variable of up to eight conditions: high blood pressure, diabetes, cancer, lung disease, heart disease, stroke, psychiatric problems, and arthritis (range 0–8), consistent with previous research (17). A modified eight-item version of the Center for Epidemiologic Studies Depression scale (CES-D) was used for the measurement of depressive symptoms. For this modified CES-D, participants reported on the extent to which in the previous week, they felt depressed, everything was an effort, sleep was restless, that he or she could not get going, lonely, that he or she enjoyed life, sad, and happy. To minimize the operational confounding of this covariate with the outcome, we removed the CES-D item that pertained to sleep. We reverse-coded the positive items and summed the remaining seven items for a count of recent depressive symptoms ranging from 0 to 7, consistent with prior research (18). While sleep apnea, which is a well-documented contributor to sleep difficulties, was not measured in this study, as a proxy for sleep apnea, we control for high body mass index (BMI), which is a known risk factor for sleep apnea (19). Body mass index was calculated according to the standard formula (weight in kilograms by the square of height in meters) from self-reported height and weight at each wave. We create a variable to signify high BMI as a score of 27 or higher, consistent with previous research, and control for this in our fully adjusted models (20).

### Statistical Analyses

Baseline sample characteristics are reported in Table 1. We computed descriptive statistics for sleep difficulty and cognitive function by year (Table 2). Analysis of variance was used to examine the associations between sleep difficulty and cognitive function during the 10-year duration, followed by post-hoc comparisons using the Tukey’s honestly significant difference test. Post-hoc comparisons were conducted between the sleep disturbance levels and cognitive impairment by year. Finally, a repeated-measures generalized linear mixed model (GLMM) with maximum likelihood estimation (21) and robust standard errors (22) examined associations between sleep difficulties and cognitive function longitudinally over time (Tables 3–5). To address our primary question of interest, each model specified the main effects for each sleep difficulty, time, and their interaction (time × sleep difficulty). As sleep difficulties may not change linearly over time between assessments, time was represented as a categorical variable in the repeated measures models with the baseline assessment in 2004 used as the reference. This analysis was repeated for each sleep difficulty, which was entered into the model with the ordinal structure from 1 (rarely/never) to 3 (most nights). All models controlled for time-varying covariates including chronic conditions, depressive symptoms, and high BMI. Several covariates including age at baseline, marital status, gender, race, Hispanic ethnicity, assets, and education were drawn from the baseline interview data. The cutoff statistical significance for retaining variables in the model was set to *p* value less than .05, and different models were compared based on Akaike’s information criterion. Model fit was assessed using the Wald statistic to test the null hypothesis, which was that the coefficients are simultaneously equal to zero. All analyses were performed using Stata software, version 16 (StataCorp, College Station, TX).

**Table 1.** Demographic Characteristics of the Sample in the First Wave (Year 1, 2004) (*n* = 16,201)

		Mean ± *SD* or *N* (%)
*Demographic and Health Characteristics*		
Age		67.51 ± 10.8
Gender	Male	8173 (42.4%)
	Female	11,107 (57.6%)
Marital status	Married	11,833 (80.4%)
	Living with partner	174 (1.2%)
	Separated	543 (3.7%)
	Divorced	315 (2.1%)
	Widowed	1844 (12.5%)
Race	White/Caucasian	15,569 (80.8%)
	Black/African American	2770 (14.4%)
	Other	938 (4.9%)
Assets	≤$55,000	4350 (26.4%
	$54,999 to $182,999	4151 (25.2%)
	$18,300 to $464,999	4037 (24.5%)
	≥$465,000	3960 (24.0%)
Hispanic ethnicity		1783 (9.2%)
Body mass index		27.4 ± 5.6
Self-rated health	Excellent	800 (7.0%)
	Very good	3241 (28.4%)
	Good	3895 (34.1%)
	Fair	2539 (22.2%)
	Poor	951 (8.3%)
Depressive symptoms (CES-D)		1.46 ± 2.0
Chronic conditions	0	3305 (17.2%)
	1	5035 (26.1%)
	2	4908 (25.5%)
	≥3	6023 (31.2%)
*Sleep Difficulty Characteristics*		
Difficulty initiating sleep	Never	5332 (51.4%)
	Sometimes	3552 (34.3%)
	Most nights	1482 (14.3%)
Nocturnal awakenings	Never	3607 (34.9%)
	Sometimes	3947 (38.3%)
	Most nights	2766 (26.8%)
Waking early	Never	5441 (52.5%)
	Sometimes	3599 (34.8%)
	Most nights	1317 (12.7%)
Feeling rested	Never	5089 (57.6%)
	Sometimes	2621 (29.7%)
	Most nights	1124 (12.7%)

*Note:* CES-D = Center for Epidemiologic Studies Depression scale.

**Table 2.** General Linear Mixed Models Examining Cognitive Function and Difficulty Initiating Sleep (*n* = 16,201)

	Model 1				Model 2				Model 3			
		95% CI				95% CI				95% CI		
	*b*	Lower	Upper	*p* Value	*b*	Lower	Upper	*p* Value	*b*	Lower	Upper	*p* Value
*Difficulty Initiating Sleep*												
Direct effect of difficulty												
Never	Reference				Reference				Reference			
Sometimes	−0.19	−0.32	−0.06	0.004	−0.19	−0.32	−0.06	0.004	−0.15	−0.28	−0.02	0.023
Most nights	−0.21	−0.39	−0.03	0.023	−0.21	−0.39	−0.03	0.023	−0.13	−0.31	0.04	0.137
Direct effect of time												
2004	Reference				Reference				Reference			
2006	−0.34	−0.43	−0.24	0.000	−0.34	−0.43	−0.24	0.000	−0.25	−0.35	−0.16	0.000
2010	−1.18	−1.28	−1.09	0.000	−1.18	−1.28	−1.09	0.000	−1.15	−1.25	−1.05	0.000
2014	−1.68	−1.78	−1.57	0.000	−1.68	−1.78	−1.57	0.000	−1.68	−1.79	−1.58	0.000
Time × Difficulty												
2006 × Sometimes	−0.03	−0.20	0.14	0.740	−0.03	−0.17	0.13	0.840	−0.03	−0.17	0.11	0.851
2010 × Sometimes	−0.02	−0.19	0.15	0.848	−0.03	−0.19	0.15	0.781	−0.05	−0.20	0.14	0.746
2014 × Sometimes	0.03	−0.15	0.20	0.763	0.03	−0.15	0.20	0.763	0.02	−0.15	0.20	0.777
2006 × Most nights	−**0.23**	−**0.45**	−**0.01**	**0.042**	−**0.21**	−**0.43**	−**0.02**	**0.044**	−0.19	−0.41	0.02	0.082
2010 × Most nights	−0.13	−0.35	0.11	0.266	−0.13	−0.36	0.10	0.266	−0.14	−0.37	0.09	0.235
2014 × Most nights	−**0.40**	−**0.64**	−**0.17**	**0.001**	−**0.40**	−**0.63**	−**0.15**	**0.001**	−**0.40**	−**0.63**	−**0.16**	**0.001**

*Notes:* Model 1: models include no covariates. Model 2: models include all demographic covariates. Model 3: models include all demographic and health covariates. Bold indicates significance less than .05.

**Table 3.** General Linear Mixed Models Examining Cognitive Function and Nocturnal Awakenings (*n* = 16,201)

	Model 1				Model 2				Model 3			
		95% CI				95% CI				95% CI		
	*B*	Lower	Upper	*p* Value	*b*	Lower	Upper	*p* Value	*b*	Lower	Upper	*p* Value
*Nocturnal Awakenings*												
Direct effect of difficulty												
Never	Reference				Reference				Reference			
Sometimes	−0.11	−0.24	0.03	.121	−0.10	−0.23	0.04	.166	−0.09	−0.22	0.05	.207
Most nights	−0.09	−0.26	0.09	.326	−0.02	−0.17	0.13	.823	0.00	−0.15	0.15	.973
Direct effect of time												
2004	Reference				Reference				Reference			
2006	**−0.39**	**−0.51**	**−0.27**	**.000**	**−0.32**	**−0.44**	**−0.20**	**.000**	**−0.31**	**−0.43**	**−0.19**	**.000**
2010	**−1.19**	**−1.32**	**−1.07**	**.000**	**−1.16**	**−1.29**	**−1.04**	**.000**	**−1.10**	**−1.22**	**−0.97**	**.000**
2014	**−1.65**	**−1.78**	**−1.52**	**.000**	**−1.65**	**−1.78**	**−1.52**	**.000**	**−1.54**	**−1.67**	**−1.41**	**.000**
Time × Difficulty												
2006 × Sometimes	0.06	−0.12	0.24	.497	0.07	−0.11	0.24	.466	0.09	−0.08	0.27	.300
2010 × Sometimes	0.06	−0.13	0.24	.542	0.03	−0.15	0.21	.746	0.05	−0.13	0.23	.594
2014 × Sometimes	−0.05	−0.23	0.14	.632	−0.06	−0.24	0.13	.552	−0.03	−0.21	0.16	.768
2006 × Most nights	0.02	−0.17	0.21	.819	0.06	−0.12	0.25	.496	0.13	−0.06	0.32	.169
2010 × Most nights	−0.10	−0.29	0.09	.315	−0.08	−0.27	0.11	.422	−0.03	−0.22	0.17	.793
2014 × Most nights	−0.19	−0.39	0.01	.065	−0.18	−0.38	0.02	.075	−0.10	−0.30	0.10	.334

*Notes:* Model 1: models include no covariates. Model 2: models include all demographic covariates. Model 3: models include all demographic and health covariates. Bold indicates significance less than .05.

**Table 4.** General Linear Mixed Models Examining Cognitive Function and Early Morning Awakenings (*n* = 16,201)

	Model 1				Model 2				Model 3			
		95% CI				95% CI				95% CI		
	*b*	Lower	Upper	*p* Value	*b*	Lower	Upper	*p* Value	*b*	Lower	Upper	*p* Value
*Early Morning Awakenings*												
Direct effect of difficulty frequency												
Never	Reference				Reference				Reference			
Sometimes	**−0.14**	**−0.26**	**−0.01**	**.036**	−0.11	−0.24	0.02	0.086	−0.09	−0.22	0.03	0.151
Most nights	**−0.23**	**−0.41**	**−0.05**	**.014**	−0.14	−0.32	0.05	0.143	−0.12	−0.30	0.06	0.208
Direct effect of time												
2004	Reference				Reference				Reference			
2006	**−0.34**	**−0.43**	**−0.24**	**.000**	**−0.27**	**−0.37**	**−0.18**	**.000**	**−0.26**	**−0.35**	**−0.16**	**.000**
2010	**−1.20**	**−1.29**	**−1.10**	**.000**	**−1.17**	**−1.27**	**−1.07**	**.000**	**−1.09**	**−1.19**	**−0.99**	**.000**
2014	**−1.63**	**−1.73**	**−1.52**	**.000**	**−1.64**	**−1.74**	**−1.53**	**.000**	**−1.50**	**−1.61**	**−1.40**	**.000**
Time × Difficulty												
2006 × Sometimes	−0.02	−0.19	0.14	.795	0.03	−0.13	0.20	.707	0.05	−0.11	0.22	.511
2010 × Sometimes	0.00	−0.17	0.17	.990	0.01	−0.16	0.18	.924	0.01	−0.16	0.18	.920
2014 × Sometimes	−0.14	−0.31	0.03	.111	−0.12	−0.29	0.05	.163	−0.10	−0.27	0.07	.263
2006 × Most nights	−0.20	−0.43	0.03	.084	−0.15	−0.38	0.08	.195	−0.06	−0.29	0.17	.596
2010 × Most nights	−0.03	−0.27	0.20	.788	−0.04	−0.28	0.19	.722	0.01	−0.23	0.24	.956
2014 × Most nights	**−0.38**	**−0.62**	**−0.13**	**.003**	**−0.36**	**−0.61**	**−0.12**	**.004**	**−0.31**	**−0.56**	**−0.07**	**.012**

*Notes:* Model 1: models include no covariates. Model 2: models include all demographic covariates. Model 3: models include all demographic and health covariates. Bold indicates significance less than .05.

**Table 5.** General Linear Mixed Models Examining Cognitive Function and Reports of Waking and Feeling Rested (*n* = 16,201)

	Model 1				Model 2				Model 3			
		95% CI				95% CI				95% CI		
	*b*	Lower	Upper	*p* Value	*b*	Lower	Upper	*p* Value	*b*	Lower	Upper	*p* Value
*Feeling Rested*												
Direct effect of difficulty frequency												
Never	Reference				Reference				Reference			
Sometimes	**−0.13**	**−0.27**	**0.01**	**.067**	−0.10	−0.23	0.04	.163	−0.09	−0.23	0.05	.196
Most nights	−0.15	−0.34	0.04	.124	−0.12	−0.31	0.07	.222	−0.11	−0.29	0.08	.275
Direct effect of time												
2004	Reference				Reference				Reference			
2006	−0.09	−0.19	0.01	.088	−0.09	−0.19	0.02	.096	−0.08	−0.18	0.02	.118
2010	**−1.00**	**−1.11**	**−0.90**	**.000**	**−1.00**	**−1.10**	**−0.90**	**.000**	**−0.95**	**−1.05**	**−0.85**	**.000**
2014	**−1.65**	**−1.75**	**−1.55**	**.000**	**−1.66**	**−1.76**	**−1.55**	**.000**	**−1.55**	**−1.65**	**−1.45**	**.000**
Time × Difficulty												
2006 × Sometimes	−0.12	−0.31	0.07	.206	−0.13	−0.32	0.06	.190	−0.09	−0.28	0.10	.369
2010 × Sometimes	**0.20**	**0.00**	**0.39**	**.046**	0.19	0.00	0.38	.051	**0.21**	**0.02**	**0.40**	**.032**
2014 × Sometimes	0.00	−0.18	0.19	.958	0.01	−0.18	0.19	.939	0.06	−0.13	0.24	.541
2006 × Most nights	−0.14	−0.39	0.11	.264	−0.15	−0.39	0.10	.246	−0.08	−0.33	0.17	.531
2010 × Most nights	−0.02	−0.26	0.23	.896	−0.01	−0.25	0.23	.923	0.04	−0.20	0.28	.750
2014 × Most nights	−0.21	−0.46	0.05	.108	−0.19	−0.44	0.06	.133	−0.12	−0.37	0.13	.344

*Notes:* Model 1: models include no covariates. Model 2: models include all demographic covariates. Model 3: models include all demographic and health covariates. Bold indicates significance less than .05.

## Results

Summary statistics for all demographic and sleep difficulty variables at baseline are given in Table 1. The average age of the sample at baseline (Year 1, 2004) was 67.51 years (*SD* = 10.8 years). Participants reported a mean total asset value of $409,730 (*SD* = 1,080,667.7). Among participants, self-reported health was rated “excellent” by 7.0%, “very good” by 28.3%, “good” by 34.1%, “fair” by 22.2%, and “poor” by 8.3%. Among participants, 80.4% were married, 80.8% were white/Caucasian, and 9.3% were Hispanic/Latino. The average BMI of the participants was 27.4. Approximately one quarter (26.1%) reported one chronic medical condition and another quarter (25.5%) reported two chronic conditions.

Regarding sleep difficulty, the majority of individuals reported “never” having difficulty initiating sleep (51.4%), followed by those reporting experiencing difficulty “some nights” (34.3%) and “most nights” (14.3%). Regarding difficulty with nocturnal awakenings, the majority responded “some nights” (38.3%), followed by “never” (34.9%) and “most nights” (26.8%). Regarding difficulty waking too early and not being able to return to bed, the majority of participants reported “never” experiencing this difficulty (52.5%), followed by “some nights” (34.8%) and “most nights” (12.7%). Regarding waking and feeling rested, the majority of the participants reported “never” waking and feeling rested (57.6%), followed by “some nights” (29.7%) and “most nights” (12.7%). Overall, the average cognitive function score was 15.6 (*SD* = 3.9).

**Figure 1.**
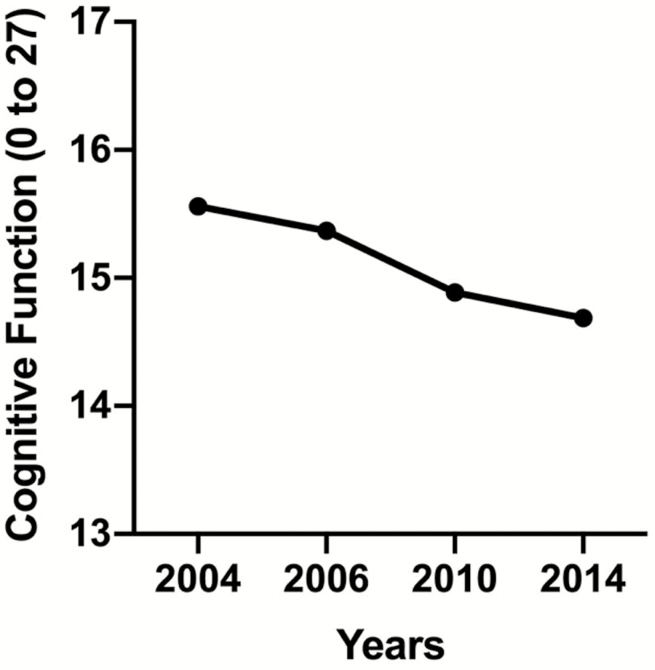
Cognitive function for a period of 10 years.

**Figure 2.**
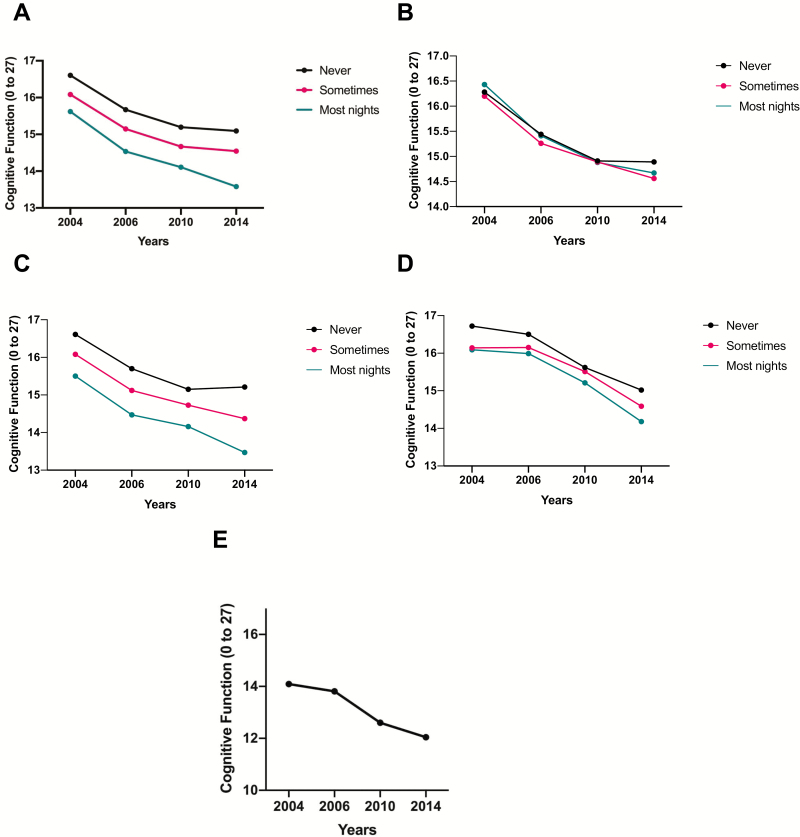
Cognitive function versus (A) trouble initiating sleep, (B) nocturnal awakenings, (C) early morning awakenings, (D) waking feeling rested, and (E) those reporting concurrent sleep difficulties for a period of 10 years.

Figure 1 displays cognitive function, which declines over time. Figure 2A–D displays wave-specific descriptive statistics for cognitive function based on each reported sleep difficulty. Overall, the figures reflect the decreasing level of cognitive function during the 10-year period.

Using analysis of variance, we found significant associations between each sleep difficulty and cognitive function (difficulty initiating sleep: *F*(2, 46,497) = 1.53, *p* < .001; nocturnal awakenings: *F*(2, 46,425) = 4.19, *p* = .015; waking too early: *F*(2, 46,480) = 229.02, *p* < .001; feeling rested: *F*(2, 35,220) = 49.94, *p* < .001). Post-hoc comparisons examining pairwise relationships using the Tukey’s honestly significant difference revealed that cognitive function was significantly different between those who “never” experienced difficulty initiating sleep and those who reported “sometimes” experiencing difficulties initiating sleep (*p* < .001) and “most nights” experiencing difficulty initiating sleep (*p* < .001). Additional post-hoc pairwise comparisons revealed that cognitive function was significantly different between those who “never” experienced nocturnal awakenings and those who experienced difficulty with nocturnal awakenings “sometimes” (*p* < .05), but not those who experienced nocturnal awakenings “most nights” (*p* = .933). Pairwise post-hoc comparisons revealed that cognitive function was significantly different between those who “never” experienced waking too early and those who experienced difficulty waking too early “sometimes” (*p* < .001) and “most nights” (*p* < .001). Post-hoc comparisons also revealed that cognitive function was significantly different between those who reported “never” waking and feeling rested and those who reported “sometimes” waking and feeling rested (*p* < .001) and those who reported “most nights” waking and feeling rested (*p* < .001).

Tables 2–5 display results of the GLMM examining the relationships between each difficulty (i.e., difficulty initiating sleep, nocturnal awakenings, early morning awakenings, and waking up feeling rested) and cognitive function over time by examining the interaction between difficulty and time. Table 2 displays the GLMM examining difficulty initiating sleep and cognitive function over time. In unadjusted Model 1 analyses, compared to “never” experiencing difficulty initiating sleep, difficulty “most nights” was associated with worse cognitive function in 2006 (*b* = −0.23, 95% confidence interval [CI]: −0.45 to −0.01, *p* < .05) and in 2014 (*b* = −0.40, 95% CI: −0.64 to −0.17, *p* < .01). In Model 2, partially adjusted analyses, compared to “never” experiencing difficulty initiating sleep, difficulty waking up too early “most nights” was associated with worse cognitive function in 2006 (*b* = −0.21, 95% CI: −0.43 to −0.02, *p* < .05) and in 2014 (*b* = −0.40, 95% CI: −0.63 to −0.15, *p* < .01). In Model 3, fully adjusted analyses, compared to “never” experiencing difficulty initiating sleep, difficulty “most nights” was associated with worse cognitive function in 2014 (*b* = −0.40, 95% CI: −0.63 to −0.16, *p* < .01). The overall test of significance suggested that the coefficients were not simultaneously equal to zero (Wald chi-square = 11,708.9, 2 degrees of freedom, *p* < .001).

Table 3 displays the GLMM examining nocturnal awakenings and cognitive function over time. Nocturnal awakenings were not, in any model, associated with cognitive function over time.

Table 4 displays the GLMM examining early morning awakenings and cognitive function over time. In Model 1, unadjusted analyses, compared to “never” waking up too early and not being able to fall back asleep, difficulty “most nights” was associated with worse cognitive function in 2014 (*b* = −0.38, 95% CI: −0.62 to −0.13, *p* < .01). In Model 2, partially adjusted analyses, compared to “never” waking up too early and not being able to fall back asleep, difficulty “most nights” was associated with worse cognitive function in 2014 (*b* = −0.36, 95% CI: −0.61 to −0.12, *p* < .01). In Model 3, fully adjusted analyses, compared to “never” waking up too early and not being able to fall back asleep, difficulty “most nights” was associated with worse cognitive function in 2014 (*b* = −0.31, 95% CI: −0.56 to −0.07, *p* < .05). The overall test of significance suggested that the coefficients were not simultaneously equal to zero (Wald chi-square = 11,218.3, 2 degrees of freedom, *p* < .001).

Table 5 displays the GLMM examining reports of waking up feeling rested and cognitive function over time. In Model 1, unadjusted analyses, compared to “never” waking up feeling rested, those who reported “sometimes” waking up feeling rested were more likely to have a higher cognitive function in 2010 (*b* = 0.20, 95% CI: 0.00 to 0.39, *p* < .05). In Model 3, fully adjusted analyses, compared to those who reported “never” waking up feeling rested, those who reported “sometimes” waking up feeling rested were more likely to have a higher cognitive function in 2010 (*b* = 0.21, 95% CI: 0.02 to 0.40, *p* < .05). The overall test of significance suggested that the coefficients were not simultaneously equal to zero (Wald chi-square = 11,021.7, 2 degrees of freedom, *p* < .001).

Table 6 displays the GLMM examining concurrent sleep difficulties and cognitive function over time. In Model 1, unadjusted analyses, compared to not having concurrent difficulties, those with these difficulties demonstrated lower cognitive function in 2010 (*b* = −0.71, 95% CI: −1.32 to −0.12, *p* < .05). In Model 2, partially adjusted analyses, compared to those not reporting concurrent difficulties, those who reported these difficulties demonstrated lower cognitive function in 2010 (*b* = −0.64, 95% CI: −1.23 to −0.05, *p* < .05). The overall test of significance suggested that the coefficients were not simultaneously equal to zero (Wald chi-square = 18,443.9, 2 degrees of freedom, *p* < .001).

**Table 6.** General Linear Mixed Models Examining Cognitive Function and Reports of Concurrent Sleep Difficulties (*n* = 16,201)

	Model 1	Model 2	Model 3									
		95% CI	*p* Value		95% CI	*p* Value	*b*	95% CI				
	*b*	Lower	Upper	*b*		Lower	Upper		*p* Value	Lower	Upper	
*Concurrent Sleep Difficulties*												
Direct effect of difficulty frequency												
Never	Reference	Reference	Reference									
Sometimes	−0.24	−0.71	0.21	.286	−0.20	−0.64	0.25	.390	−0.19	−0.63	0.26	.416
Direct effect of time												
2004	Reference	Reference	Reference									
2006	−0.35	−0.43	−0.28	.088	**−0.28**	**−0.34**	**−0.21**	**.000**	**−0.25**	**−0.32**	**−0.18**	**.000**
2010	**−1.18**	**−1.26**	**−1.11**	**.000**	**−1.16**	**−1.23**	**−1.09**	**.000**	**−1.08**	**−1.15**	**−1.01**	**.000**
2014	**−1.71**	**−1.75**	**−1.63**	**.000**	**−1.71**	**−1.79**	**−1.64**	**.000**	**−1.55**	**−1.65**	**−1.65**	**.000**
Time × Difficulty												
2006 × Concurrent difficulties	−0.36	−0.93	0.20	.197	−0.11	−0.66	0.49	.671	0.05	−0.49	0.60	.834
2010 × Concurrent difficulties	**−0.71**	**−1.32**	**0.12**	**.019**	**−0.64**	**−1.23**	**−0.05**	**.034**	−0.58	−1.17	0.01	.054
2014 × Concurrent difficulties	−0.58	−1.22	0.05	.079	−0.53	−1.17	0.10	.099	−0.50	−1.13	0.13	.123

*Notes:* Model 1: models include no covariates. Model 2: models include all demographic covariates. Model 3: models include all demographic and health covariates. Bold indicates significance less than .05.

## Discussion

We examined specific sleep difficulties and their relationship to cognitive function during a 10-year period using nationally representative, longitudinal data collected among U.S. adults aged 51 or older between 2004 and 2014. Findings from our study demonstrate that, after adjusting for demographic and health condition covariates, two of the four sleep difficulties measured in this study were associated with cognitive function over time. First, early morning awakenings were associated with worse cognitive function over time. Specifically, for those individuals who reported experiencing early morning awakenings “most nights,” compared to never reporting such difficulties, there was a significant decline in scores on the validated instruments assessing cognitive function. Second, in adjusted analyses, those who reported feeling rested “some nights” had better cognitive function over time. With an aging population in the United States, understanding the contributing factors to cognitive impairment and the initiation and worsening of AD is imperative (5). Results from our study offer clinical and practical utility in identifying the specific sleep difficulties that relate to cognitive function.

Our results extend and refine the published literature on sleep and human aging. Our research is consistent with cross-sectional data which has shown that older adults reporting either insomnia symptoms or late-life insomnia exhibit significantly reduced performance in learning rate and in temporal order judgment tests (6), as well as research that has documented associations between objective, wrist-worn actigraphy recordings of poor sleep (i.e., increased fragmentation) and cognitive decline (4,5,7). Also, our findings on the relationship between sleep difficulties and cognitive function are confirmatory with meta-analysis documenting a strong association between both poor sleep quality and insomnia and greater risk for AD (8). However, findings from research employing both objective and subjective measures of sleep difficulties, has been mixed. Some work has found an association between both self-reported measures (i.e., poor sleep quality) and objective measures (i.e., actigraphy recorded sleep difficulties) of sleep difficulty and cognitive decline (4), but other work has found no relationship between self-reported measures, only between objective measures of sleep difficulty and cognitive function (5). It could be that each sleep difficulty offers a novel contribution or risk factor for cognitive decline. Therefore, retaining the individual difficulties in our analyses may have enabled new insights into distinct sleep difficulties that are most important for cognitive function, which would be challenging to detect with summary scores as have been previously employed in the literature.

There are several mechanisms that may aid in the interpretation of our findings. First, without restorative, sufficient sleep older adults reporting difficulties with early awakenings or not feeling refreshed could have been deprived of the restoration of energy stores for which sleep is known. Second, memory consolidation takes place during sleep (23). Without restorative sleep, this ability is likely deprived, leading to worse performance on the validated cognitive batteries employed in this study. Third, research has uncovered the beneficial role sleep plays in removing dementia and AD biomarkers (24). Finally, while a shift in circadian preference toward earlier rising times is documented in the literature as a normal part of age-related neurological processes (25), it could be that our research highlights a potentially greater decline in cognitive function among those who experience an extreme shift in their rising time.

Also, it is interesting that we find reports of waking up feeling rested “some nights” were associated with better cognitive function over time. Certainly, feeling well-rested is indicative of the absence of sleep difficulty, suggesting a different perspective on the relationship between sleep and cognitive function. It should be noted, however, that it is somewhat surprising to see the relationship between waking up feeling rested “sometimes” but not “most nights.” One plausible explanation for this finding is that even a healthy sleeper may experience occasional difficulty and associated subsequent low feelings of being rested upon waking from time to time, leading most individuals to report “sometimes” waking and feeling rested as opposed to “most nights.” Another plausible explanation for the relationship between waking up feeling rested “sometimes” and cognitive impairment may simply have to do with unbalanced groups, for the majority of the sample reported “never” waking and feeling rested at baseline (57.6%), followed by approximately one third who reported “sometimes” (29.7%) and a significantly smaller proportion reporting waking and feeling rested “most nights” (12.7%). In either case, our results showed that, even in fully adjusted models, this effect persists.

Although we included a broad array of sleep difficulties in our analysis (i.e., difficulty initiating sleep, nocturnal awakenings, early morning awakenings, and waking up feeling rested), only early morning awakenings and waking up feeling rested were significantly associated with cognitive function over time. The lack of significant findings for the other sleep difficulty items may be understood through the lens of previous literature. First, extensive literature documents that sleep is fundamentally more challenging among older adults compared to their younger counterparts (26). Thus, lifespan-related decay in sleep may be difficult to disentangle from difficulties that may determine the worsening of cognition. Also, research has shown that medical comorbidities among older adults may limit sleep capabilities (27). Specifically, research has shown that, through comprehensive screening to recruit physically and psychologically healthy older adults, insomnia prevalence among older adults is quite low (between 2% and 3%) (28), suggesting that we may have had different findings if we performed our analysis on adults who remained healthy throughout the study. Alternatively, while we control for various evidence-based demographic factors and health conditions, it is possible that these variables may be in the causal pathway between sleep disturbance and cognitive functioning (e.g., sleep disruption may lead to depressive symptoms which then cause reductions in cognitive functioning). Therefore, models with full covariate adjustment should be interpreted cautiously. Another possible explanation may have to do with sleep state misperception. Specifically, research using multiple methods of sleep, including polysomnography and diary reports, found significant night-to-night variability in sleep parameters within older individuals, suggesting some individuals are simply poor judges of sleep depth or quality (29).

Sleep difficulty is more prevalent among older adults than younger adults (30) and in prior studies has been associated with AD and dementia. Furthermore, previous literature has shown that the proportion of older adults who reported sleep difficulty at baseline nearly doubled after 2 years (27).

### Strengths and Limitations

The longitudinal design of the HRS data is a significant strength of this study. They enabled our analysis of cognitive function and sleep difficulty over time. Cross-sectional studies, by comparison, are unable to address the core question of this study that examined the variability in cognitive function due to specific sleep difficulties over time. Unfortunately, while the HRS has been collecting data for nearly one decade prior to the waves selected for this study, questions on sleep were not included until 2004. Furthermore, beginning in the 2006 wave of data collection, the sleep measures were offered only in alternate waves, making for uneven follow-up intervals.

The sleep difficulty measures employed in this study included simplistic measures of frequency (e.g., “never,” “sometimes,” and “most nights”). Also, previous research that has examined self-reported sleep difficulty and objectively recorded sleep (i.e., wrist-worn actigraphy (31) and polysomnography) has shown weak correlations between self-reported sleep difficulty questionnaire responses and objective measures of sleep. Another notable limitation is that the self-reported measures are prone to bias among all groups but particularly among older adults who may be affected by cognitive decline and memory impairment which compromise their ability to offer an accurate rendering of the nature and frequency of sleep difficulties.

An additional limitation is that our regression models used time as a categorical predictor rather than a continuous one. Based on the trends of cognition over time (shown in Figure 2), we did not believe that the relationship between time and cognition was linear. To account for this, time was coded as a categorical variable and all main and interaction effects of time were interpreted with 2004 as the reference. As a result, the interpretation of the sleep difficulty by time interaction is more complex. However, we believe that the additional complexity of these models makes them more accurate. Future analyses should examine whether the trends shown in the current data are maintained at later assessment periods. A related limitation is that the current analysis uses multiple comparisons. The goal of the current analysis is to examine multiple components of sleep disruption, time, and their interaction effects using multiple levels of covariate adjustment. This requires the interpretation of multiple effects. Using methods to adjust for multiple comparisons (e.g., Bonferroni correction) might lead to ignoring moderate effect-size associations. Additional research is required to replicate the findings shown in the current study. Finally, obstructive sleep apnea may contribute to sleep difficulties, such as early morning awakenings, yet was not provided in this data set. As a proxy for sleep apnea, we control for high BMI, which is a known risk factor for sleep apnea (19). Future research may consider including this condition as a covariate in an exploration of the relationship between sleep difficulty and cognitive function.

### Implications and Future Research

Several consensus statements call for increased attention to nature and methods for attenuating sleep difficulties among older adults, including a National Institutes of Health consensus statement, which recently addressed the diagnosis, risks, consequences, and treatment of chronic insomnia among adults (32). The American Academy of Sleep Medicine has published practice guidelines for evaluating and managing sleep difficulties. The current study examined sleep difficulties and cognitive function among older adults, with the aim of informing policies and programs for improving the key aspects of sleep associated with cognitive function and the ultimate aim of improving cognitive function among older adults. Research using data from the Established Populations for the Epidemiologic Studies of the Elderly suggests that treating sleep difficulty among older adults may be a promising approach for decelerating the rate of cognitive decline (33).

Our results also identify a number of opportunities for future research. For instance, research to better identify the causal pathway between sleep difficulty and the onset and development of AD and related dementia is one opportunity for future research. Future research may also consider how to design programs to attenuate sleep difficulty among older adults. For instance, evidence-based programs such as Cognitive Behavioral Therapy for Insomnia (34) may be tailored to the needs of older adults.

## Conclusions

Sleep disturbance is more prevalent among older adults relative to their younger counterparts and is associated with cognitive decline and risk for AD. We used a large, national data set to examine sleep difficulties and cognitive function across a 10-year period among older adults in the United States. Our results show that early morning awakenings were associated with worse cognitive function, whereas reports of waking up feeling rested were associated with better cognitive function, over time. Future research may examine methods for attenuating sleep difficulties among older adults.
